# Association of Objective Sleep Measures and White Matter Limbic System Integrity in Community‐Dwelling Cognitively Unimpaired Older Black and White Adults

**DOI:** 10.1002/alz.085383

**Published:** 2025-01-03

**Authors:** Omonigho Michael Bubu, Joshua L Gills, Oliver Cesar, Zanetta Kovbasyuk, Anamarie Sogade, Joanna Dominguez, Laurel Y Browne, Elena Valkanova, Alfred Mbah, Luisa F Figueredo, Korey Kam, Anna E Mullins, Ankit Parekh, Arjun V. Masurkar, Andrew Varga, Ricardo Osorio

**Affiliations:** ^1^ NYU Grossman School of Medicine, New York, NY USA; ^2^ University of South Florida, Tampa, FL USA; ^3^ Icahn School of Medicine at Mount Sinai, New York, NY USA

## Abstract

**Background:**

We examined racial differences between measures of limbic white matter tracts and objective sleep parameters in cognitively unimpaired older‐adults.

**Method:**

This cross‐sectional study included 170 community‐dwelling cognitively unimpaired older‐adults (mean±SD: age = 67.2±5.2y) participating in NYU studies of sleep, aging, and memory. Subjects completed polysomnography (NPSG) and brain MRI. Sleep measures of interest included total sleep time (TST), NREM stages 2, 3 and REM sleep duration, sleep latency and fragmentation, slow sleep spindles (SSP), sleep spindle density (SPD), AHI4% and REM AHI4%. Microstructural properties of the cingulum, uncinate fasciculus (UF), and fornix were estimated using diffusional tensor imaging (DTI) metrics including radial kurtosis (RK), radial diffusivity (RD), and fractional anisotropy (FA). Adjusted Linear mixed‐effects regression models examined associations between race, sleep, race*sleep and DTI metrics.

**Result:**

Participants were 44.1% Black with 16.8±2.5y of education. Subjects were relatively matched on AHI4%, age, BMI and educational level. In the cingulum, increased fragmentation was associated with higher RD β[Left] = 0.0026, *p* = 0.022), decreased RK (β[Right] = ‐0.0069, *p* = 0.039) in Blacks. Additionally, increased sleep latency was associated with reduced FA (β[Right] = ‐0.0010, *p* = 0.019), higher RD (β [Right] = 0.0010, *p* = 0.008) in Blacks. In the UF, increased fragmentation was associated with higher RD(β[Left] = 0.0052, *p* = 0.019) and lower RK (β[Right] = ‐0.0010, *p* = 0.039) in Blacks; increased AHI4%REM was associated with reduced FA β[Left] = 0.0007, *p* = 0.041), higher RD (β[Right] = ‐0.0007, *p* = 0.027) and lower RK (β[AHI4%, Left] = ‐0.0029, *p* = 0.001) in Blacks. Furthermore, higher SSP (βRight] = 0.00031, *p* = 0.045 and SPD (β[Right] = 0.045, *p* = 0.025) was associated with higher RK in Blacks. In the cingulum of white participants, higher AHI4%REM was associated with reduced FA β[right] = ‐0.0006, *p* = 0.013), higher RD β[Left] = 0.0005, *p* = 0.051), and reduced RK β[Right] = ‐0.00198, *p* = 0.008). Additionally, NREM stage‐2 sleep duration was associated with higher FA β[Right] = 0.0010, *p* = 0.037) and higher RK β[Right] = 0.0031, *p* = 0.040). In the UF, increased fragmentation was associated with reduced RK β[Left] = ‐0.017, *p* = 0.047) in Whites.

**Conclusion:**

Cognitively‐unimpaired Black and White older‐adults showed sleep disruption associations with limbic white matter integrity, with race‐specific associations observed across various microstructural properties of the cingulum, UF, and fornix. Thus, highlighting possible sleep‐related underpinnings of racial differences in Alzheimer disease‐risk.

